# Thiamine administration in septic shock: a post hoc analysis of two randomized trials

**DOI:** 10.1186/s13054-024-04818-1

**Published:** 2024-02-06

**Authors:** Jacob Vine, John H. Lee, Max S. Kravitz, Anne V. Grossestreuer, Lakshman Balaji, Shannon B. Leland, Noa Berlin, Ari Moskowitz, Michael W. Donnino

**Affiliations:** 1 Center for Resuscitation Science, Beth Israel Deaconess Medical Center, Boston, MA USA; 2 Department of Emergency Medicine, Beth Israel Deaconess Medical Center, Boston, MA USA; 3 Department of Anesthesiology, Critical Care and Pain Medicine, Boston Children’s Hospital, Boston, MA USA; 4 Cummings School of Veterinary Medicine at Tufts University, North Grafton, MA USA; 5 Division of Critical Care Medicine, Montefiore Medical Center, The Bronx, NY USA; 6 Bronx Center for Critical Care Outcomes and Resuscitation Research, The Bronx, NY USA; 7 Division of Pulmonary, Critical Care, and Sleep Medicine, Beth Israel Deaconess Medical Center, Boston, MA USA

**Keywords:** Sepsis, Shock, Thiamine, Kidney injury, Renal protective

## Abstract

**Background:**

This is a post hoc analysis of combined cohorts from two previous Phase II clinical trials to assess the effect of thiamine administration on kidney protection and mortality in patients with septic shock.

**Methods:**

Patient-level data from the Thiamine in Septic Shock Trial (NCT01070810) and the Thiamine for Renal Protection in Septic Shock Trial (NCT03550794) were combined in this analysis. The primary outcome for the current study was survival without the receipt of renal replacement therapy (RRT). Analyses were performed on the overall cohort and the thiamine-deficient cohort (thiamine < 8 nmol/L).

**Results:**

Totally, 158 patients were included. Overall, thiamine administration was associated with higher odds of being alive and RRT-free (adjusted odds ratio [aOR]: 2.05 [95% confidence interval (CI) 1.08–3.90]) and not needing RRT (aOR: 2.59 [95% CI 1.01–6.62]). In the thiamine-deficient group, thiamine administration was associated with higher odds of being alive and RRT-free (aOR: 8.17 [95% CI 1.79–37.22]) and surviving to hospital discharge (aOR: 6.84 [95% CI 1.54–30.36]). There was a significant effect modification by baseline thiamine deficiency for alive and RRT-free (interaction, *p* = 0.016) and surviving to hospital discharge (*p* = 0.019).

**Conclusion:**

In the combined analysis of two previous randomized trials, thiamine administration was associated with higher odds of being alive and RRT-free at hospital discharge in patients with septic shock. This signal was stronger in patients with thiamine deficiency.

**Supplementary Information:**

The online version contains supplementary material available at 10.1186/s13054-024-04818-1.

## Introduction

Thiamine has been proposed as a mitochondrial resuscitator that may attenuate organ injury and lessen mortality in septic shock [1, 2]. Kidney injury is frequently seen in patients with septic shock and has been associated with poor clinical outcomes, including longer length of intensive care unit (ICU) stay and higher mortality [3]. Kidney injury in septic shock has been traditionally ascribed to renal hypoperfusion from cytokine-mediated vasodilation, ultimately resulting in tubular necrosis and renal failure [4, 5]. However, studies have shown that kidney injury can occur even in the absence of prolonged hypoperfusion. The histopathologic pattern of kidney injury in sepsis often features apoptosis, hinting at alternative mechanisms beyond impaired oxygen delivery, such as mitochondrial dysfunction [6].

Thiamine is a cofactor for pyruvate dehydrogenase and critical for aerobic respiration [7, 8]. Thiamine is also a necessary component of the pentose phosphate pathway, which plays a role in reducing oxidative stress [9, 10]. Thiamine deficiency has been associated with organ dysfunction and has been studied in patients with septic shock [10–13]. Prior studies from our group have explored thiamine as a renal protective agent in sepsis [1, 2]. Although these studies showed promising results with point estimates favoring thiamine supplementation, the relatively small sample sizes of the trials may have led to type 2 error. In addition, there have been several other studies [14–16] that have evaluated thiamine supplementation in septic shock; they did not specifically investigate renal outcomes or focus on baseline thiamine levels.

In this study, we pooled patient-level data from our two prior randomized control trials (RCTs) of thiamine supplementation in septic shock [1, 2] to test the hypotheses that (1) thiamine supplementation improves the odds of being alive and renal replacement therapy (RRT) free at the time of discharge and (2) thiamine supplementation has greater benefit in those with thiamine deficiency.

## Methods

### Setting and patients

The cohort for the present post hoc analysis is drawn from two previous Phase II clinical trials. The Thiamine in Septic Shock Trial (TSS) (NCT01070810) enrolled patients from two urban academic centers between 2010 and 2014 [1]. The Thiamine for Renal Protection in Septic Shock Trial (TRPSS) (NCT03550794) enrolled patients from three urban academic centers between 2015 and 2021 [2]. Patients in the TSS study were included in the present study if they were not already receiving RRT at the time of enrollment. All patients in the TRPSS trial were included. Differences between trials with respect to inclusion/exclusion criteria, the intervention, and outcomes measured can be found in the original trial publications [1, 2]. Both RCTs were approved by local Institutional Review Boards and overseen by Data Safety and Monitoring Boards.

### Exposure and outcomes

The primary exposure for the present study was randomization group (thiamine administration vs. placebo). The primary outcome was the composite of being alive and RRT-free at the time of hospital discharge. Secondary outcomes included in-hospital survival, receipt of RRT, and changes in serum creatinine and lactate levels between enrollment and 72 h after enrollment.

### Statistical analysis

A description of the baseline characteristics is presented by the treatment group. Categorical variables are summarized by frequencies and percentages. Percentages are calculated according to the number of patients for whom data are available. Continuous variables are summarized using means (standard deviations, SD) or medians (interquartile range, IQR) based on the distribution of the data.

For the primary composite outcome of alive and RRT-free, we performed a logistic regression analysis with the outcome as the dependent variable with treatment group (thiamine or placebo) and RCT as independent variables. All binary outcomes were analyzed similarly. Results are described as an adjusted odds ratio (aOR) with a 95% confidence interval (CI). A post hoc sensitivity analysis additionally included baseline SOFA score to adjust for illness severity.

For the continuous outcomes of serum creatinine level and serum lactate level, we constructed linear mixed models to account for the longitudinal nature of the data. We compared repeated measures of the laboratory values at each time point (0 h [baseline value], 24 h, 48 h, 72 h) between arms with independent variables of treatment group, time, the interaction between time and treatment group, and which study the patient was enrolled in, with an independent covariance structure. If a patient died or received RRT before any time point, creatinine levels were imputed by carrying forward the last known value before the first occurring event with a 20% penalty. For lactate levels, a 20% penalty was assigned to the last known value prior to death if a patient died before the time point. Results are described as a geometric mean difference with a 95% CI at 72 h. Lactate and creatinine values were log-transformed for this analysis due to substantial deviation from a normal distribution.

To assess heterogeneity of treatment effect based on baseline thiamine deficiency (plasma/serum thiamine < 8 nmol/L), an interaction term was added to the logistic regression model for the primary outcome for randomization group*deficiency status. Statistical analyses were performed using Stata 18.0 (StataCorp, College Station, TX). Significance was a priori set at *p* < 0.05.

## Results

### General cohort

A total of 158 patients were included, with pooled demographic and baseline data in Table 1. There were no statistically significant differences between groups with regards to these characteristics.

**Table 1:** Baseline characteristics

	Overall			Thiamine < 8 nmol/L		
Variable	Total (*n* = 158)	Thiamine (*n* = 73)	Placebo (*n* = 85)	Total (*n* = 46)	Thiamine (*n* = 19)	Placebo (*n* = 27)
*Demographics*						
Age (median, IQR)	70 (60, 79)	72 (60, 82)	70 (60, 78)	65 (57, 78)	67 (52, 78)	65 (58, 75)
Female, *n* (%)	73 (46%)	37 (51%)	36 (42%)	24 (52%)	9 (47%)	15 (56%)
*Race, n (%)*						
Black/African American	14 (9%)	6 (8%)	8 (9%)	5 (11%)	2 (11%)	3 (11%)
White	120 (76%)	56 (77%)	64 (75%)	32 (70%)	14 (74%)	18 (67%)
Unknown/other	24 (15%)	11 (15%)	13 (15%)	9 (20%)	3 (16%)	6 (22%)
BMI (median, IQR)	27.9 (24.2, 34.3)	28.3 (23.8, 34.6)	27.6 (24.7, 34.3)	27.9 (24.8, 34.2)	28.1 (22.1, 32.7)	27.5 (25.2, 36.0)
*Past medical history*						
CAD, *n* (%)	32 (20%)	14 (19%)	18 (21%)	6 (13%)	3 (16%)	3 (11%)
CHF, *n* (%)	28 (18%)	11 (15%)	17 (20%)	6 (13%)	1 (5%)	5 (19%)
Dementia, *n* (%)	10 (6%)	6 (8%)	4 (5%)	5 (11%)	3 (16%)	2 (7%)
Diabetes, *n* (%)	50 (32%)	27 (37%)	23 (27%)	12 (26%)	6 (32%)	6 (22%)
Pulmonary disease/COPD, *n* (%)	20 (13%)	11 (15%)	9 (11%)	3 (7%)	1 (5%)	2 (7%)
CKD, *n* (%)	28 (18%)	14 (19%)	14 (16%)	8 (17%)	4 (21%)	4 (15%)
*Laboratory values, interventions, and illness severity*						
Creatinine (median, IQR)	1.9 (1.4, 2.7)	1.9 (1.2, 2.5)	2.0 (1.5, 2.7)	1.7 (1.1, 3.0)	1.2 (1.0, 3.0)	1.8 (1.2, 3.0)
Lactate (median, IQR)	3.4 (2.5, 4.7)	3.1 (2.4, 4.5)	3.4 (2.6, 4.7)	3.2 (2.6, 4.5)	2.9 (2.3, 4.1)	3.4 (2.6, 4.7)
Vasopressor administered	158 (100%)	73 (100%)	85 (100%)	46 (100%)	19 (100%)	27 (100%)
SOFA score (mean ± SD)	10.6 ± 3.7	10.3 ± 3.6	10.8 ± 3.8	10.2 ± 3.7	10.1 ± 3.4	10.2 ± 4.0
Mechanical ventilation, *n* (%)	101 (64%)	49 (67%)	52 (61%)	30 (65%)	14 (74%)	16 (59%)

Definition of abbreviations: BMI = body mass index; CAD = coronary artery disease; CHF: congestive heart failure; COPD: chronic obstructive pulmonary disease; CKD = chronic kidney disease; SOFA = sequential organ failure assessment

### Primary outcomes

**Fig. 1:**
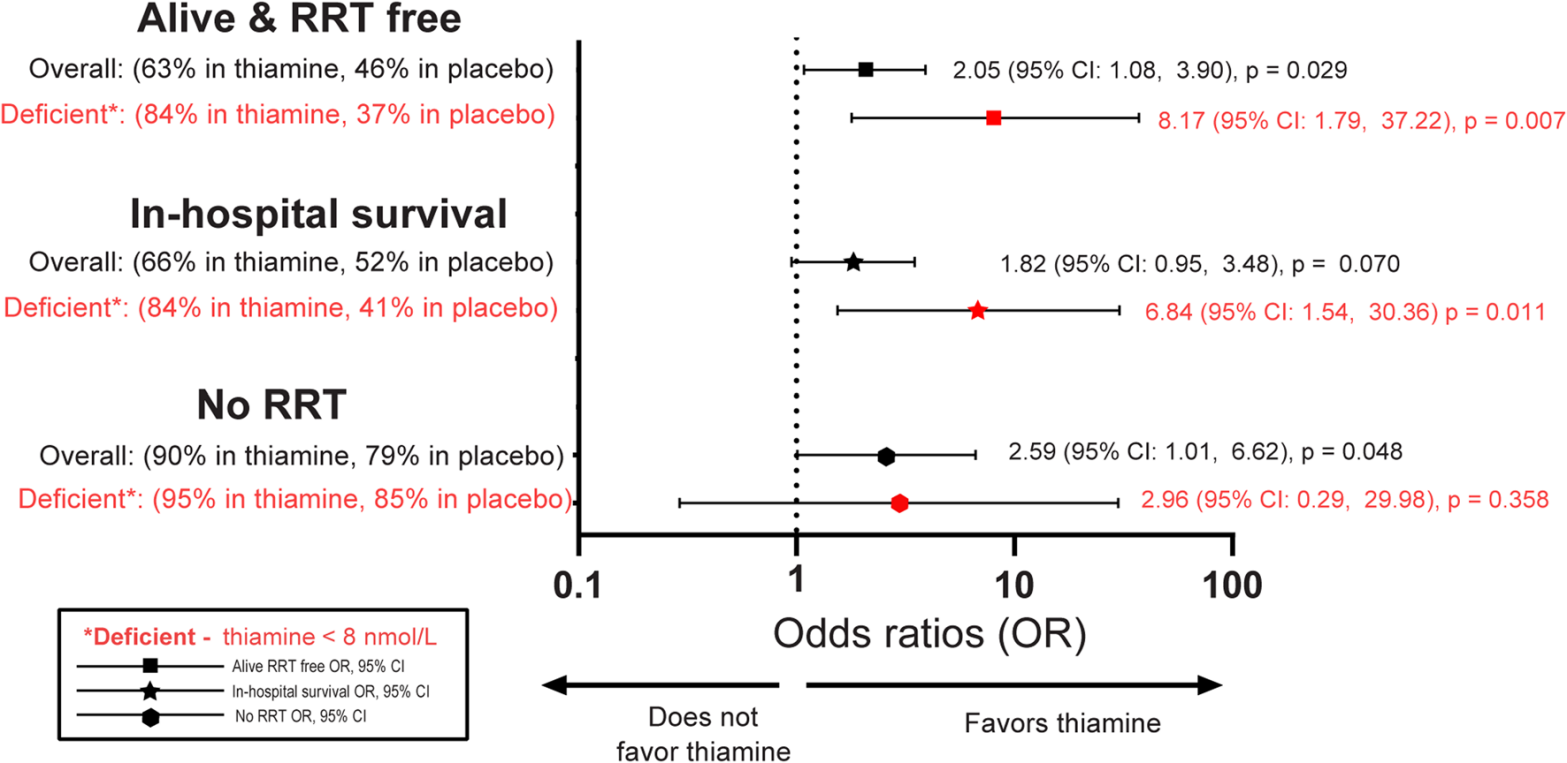
Forest plot of odds ratio between thiamine vs. placebo administration for the primary outcome and secondary outcomes, in-hospital survival and no RRT. The odds ratio on the x-axis is in log-scale. RRT = renal replacement therapy. CI = confidence interval

The primary outcome of alive and RRT-free occurred in 46 (63%) of the thiamine intervention group and 39 (46%) in the control group. Patients receiving thiamine had higher odds of being alive and RRT-free at discharge (aOR 2.05 [95% CI 1.08–3.90], *p* = 0.029). A total of 46 (29%) patients had low baseline thiamine levels. In the thiamine-deficient patients, patients receiving thiamine had higher odds of being alive and RRT-free at discharge (aOR: 8.17 [95% CI 1.79–37.22]; *p* = 0.007). These results are shown in Fig. 1. Additional outcomes by intervention group are found in Fig. 1 and Table 2. A post hoc sensitivity analysis additionally controlling for baseline SOFA found near-identical results. The results for the patients without thiamine deficiency can be found in the data supplement (Additional file 1: Tables S1, S2).

**Table 2:** Summary of outcomes for overall and thiamine-deficient cohort

Overall				
	Thiamine (*n* = 73)	Placebo (*n* = 85)	Geometric mean difference* (95% CI)	*p* Value
*Serum creatinine (median (IQR), mg/dL)*				
Enrollment	1.9 (1.2, 2.5)	2.0 (1.5, 2.7)	0.87 (0.71–1.06)	0.165
24 h	1.8 (1.0, 2.8) n = 72	2.2 (1.3, 3.1) n = 83	0.82 (0.68–0.99)	0.044
48 h	1.7 (0.9, 2.8) n = 69	2.0 (1.2, 3.3) n = 83	0.79 (0.65–0.95)	0.015
72 h	1.4 (0.8, 2.4) n = 68	2.0 (1.1, 3.1) n = 79	0.77 (0.64–0.94)	0.010
*Serum lactate (mmol/L, median, IQR)*				
Enrollment	3.1 (2.4, 4.5)	3.4 (2.6, 4.7)	1.00 (0.81–1.25)	0.965
24 h	2.0 (1.4, 2.9) n = 71	2.5 (1.6, 4.4) n = 84	0.84 (0.67–1.04)	0.101
48 h	1.8 (1.2, 2.6) n = 65	2.0 (1.5, 3.1) n = 83	0.88 (0.71–1.09)	0.252
72 h	1.6 (1.1, 2.6) n = 64	1.9 (1.4, 2.6) n = 80	0.90 (0.72–1.11)	0.323
In-hospital survival, *n* (%)	48 (66%)	44 (52%)	aOR: 1.82 (0.95–3.48)	0.070
No RRT, *n* (%)	66 (90%)	67(79%)	aOR: 2.59 (1.01–6.62)	0.048
Alive and RRT-free, *n* (%)	46 (63%)	39 (46%)	aOR: 2.05 (1.08–3.90)	0.029

Thiamine < 8 nmol/L				
	Thiamine (*n* = 19)	Placebo (*n* = 27)	Geometric mean difference (95% CI)	*p* Value
*Serum creatinine (median (IQR), mg/dL)*				
Enrollment	1.2 (1.0, 3.0)	1.8 (1.2, 3.0)	0.77 (0.51–1.14)	0.193
24 h	1.1 (0.8, 2.8)	2.0 (1.3, 3.1) n = 26	0.70 (0.47–1.04)	0.081
48 h	1.3 (0.7, 2.3)	2.0 (1.1, 3.1) n = 26	0.67 (0.45–0.99)	0.047
72 h	0.9 (0.6, 2.0) n = 17	2.0 (1.1, 3.0) n = 25	0.70 (0.47–1.04)	0.078
*Serum lactate (mmol/L, median, IQR)*				
Enrollment	2.9 (2.3, 4.1)	3.4 (2.6, 4.7)	0.86 (0.63–1.18)	0.356
24 h	1.7 (1.4, 2.5)	1.9 (1.5, 3.5) n = 26	0.75 (0.55–1.02)	0.069
48 h	1.7 (1.3, 2.6) n = 16	1.9 (1.5, 2.4) n = 26	0.73 (0.53–1.01)	0.055
72 h	1.6 (1.1, 2.5) n = 16	1.9 (1.3, 2.9) n = 25	0.67 (0.49–0.92)	0.015
In-hospital survival, *n* (%)	16 (84%)	11 (41%)	aOR: 6.84 (1.54–30.36)	0.011
No RRT, *n* (%)	18 (95%)	23 (85%)	aOR: 2.96 (0.29–29.98)	0.358
Alive and RRT-free, *n* (%)	16 (84%)	10 (37%)	aOR: 8.17 (1.79–37.22)	0.007

*Geometric mean difference < 1 indicates that outcome is lower in the thiamine group. For serum creatinine and lactate, different n is due to missing data. Imputed values used in 18 patients for RRT and 9 for death. Definition of abbreviations: CI = confidence interval; IQR = interquartile range; RRT = renal replacement therapy

### Heterogeneity of treatment effect

There was a significant effect modification of the primary outcome, alive and RRT-free, by baseline thiamine deficiency (interaction, *p* = 0.016) and with the key secondary outcome in-hospital survival (*p* = 0.019) but not with regards to RRT (*p* = 0.901).

## Discussion

In this post hoc analysis of two RCTs, thiamine administration resulted in a greater proportion of patients who were alive and RRT-free compared to placebo. Thiamine administration was also found to improve several secondary outcomes. Significant heterogeneity of treatment effect was seen, such that the effects of thiamine were greater in patients with thiamine deficiency.

Recently, three other RCTs have investigated the effect of thiamine administration in septic shock [14–16]. In all three studies, thiamine administration resulted in no difference in mortality, although thiamine administration was associated with significant lactate reduction in the study by Petsakul et al. [15]. It is worth noting that the proportion of patients with thiamine deficiency in the studies by Petsakul et al. and Pereira et al. [16] was significantly lower than the 26% in of patients who were thiamine-deficient in the present cohort (Harun et al. did not report baseline thiamine levels). The lower rate of thiamine deficiency in RCTs not included in the present study may be due to different approaches to predictive enrichment, alternative measures of thiamine level, or a combination thereof. In addition, the three other RCTs described were not focused on kidney outcomes, included patients with end-stage kidney disease, and did not provide a comprehensive assessment of acute kidney injury as was the focus in the present trial. Differential rates of thiamine deficiency and different inclusion criteria may explain differences in results of the present study and existing RCTs.

A component network meta-analysis by Fujii et al., assessing the effect of vitamin C, thiamine, and glucocorticoids on long-term mortality in septic shock found possible harm with thiamine treatment [17]. This study analyzed trials with multiple treatments and assumed an additive effect in order to estimate the effects of individual treatments. However, this finding is inconsistent with thiamine-only trials in septic shock and does not include data from the TRPSS trial [2, 10, 14–16]. Further, the network meta-analysis suffers from the same considerations outlined in the preceding paragraph—namely it does not focus on a thiamine-deficient population, does not exclude patients with pre-existing ESRD, and does not comprehensively describe kidney-specific outcomes.

This study has several limitations. First, the analysis was post hoc and excluded some patients with baseline ESRD from the TSS trial. Second, plasma thiamine levels were measured for the analyzed trials in this study. Given the heterogeneity of treatment effect seen by thiamine level, it is clear that this approach provides clinically relevant data regarding thiamine deficiency status. Other approaches to thiamine measurement have been previously described, most notably the measurement of thiamine diphosphate from whole blood, and should be further explored in future studies. In addition, rapid assessment of thiamine level is currently not feasible. An important future work includes investigating the feasibility of predicting thiamine deficiency using other clinically available data as a predictive enrichment strategy for future clinical trials of thiamine in septic shock.

## Conclusion

In this post hoc analysis of two RCTs, intravenous thiamine administration was associated with a greater proportion of patients who were alive and RRT-free compared to placebo. The signal for benefit was stronger in the group of patients who had low thiamine levels.

## Supplementary Information

**Additional file 1.** Baseline characteristics and results for patients without thiamine deficiency.

## Data Availability

The datasets used and/or analyzed during the current study are available from the corresponding author on reasonable request.
