# Supplementary material for: Thiamine administration in septic shock: a post hoc analysis of two randomized trials
Source: Crit Care. 2024 Feb 6;28:41. doi: 10.1186/s13054-024-04818-1 (PMC10845751; doi:10.1186/s13054-024-04818-1)
Supplement: Supplementary file 1 — Additional file 1. Baseline characteristics and results for patients without thiamine deficiency. [file 13054_2024_4818_MOESM1_ESM.docx]

Table S1. Baseline characteristics for non-thiamine deficient cohort.

|  | **Thiamine >=8** | | |
| --- | --- | --- | --- |
| **Variable** | **Total (n=106)** | **Thiamine (n=52)** | **Placebo (n=54)** |
| **Demographics** | | | |
| Age (median, IQR) | 71 (60, 82) | 74 (62, 84) | 70 (60, 78) |
| Female, n (%) | 47 (44%) | 25 (48%) | 22 (41%) |
| **Race, n (%)**  Black/African American  White  Unknown/Other | 9 (8%)  83 (78%)  14 (13%) | 4 (8%)  40 (77%)  8 (15%) | 5 (9%)  43 (80%)  6 (11%) |
| BMI (median, IQR)* | 27.9 (24.0, 34.1) | 28.5 (24.6, 34.6) | 27.6 (23.6, 34.0) |
| **Past Medical History** | | | |
| CAD, n (%) | 24 (23%) | 9 (17%) | 15 (28%) |
| CHF, n (%) | 20 (19%) | 9 (17%) | 11 (20%) |
| Dementia, n (%) | 4 (4%) | 3 (6%) | 1 (2%) |
| Diabetes, n (%) | 35 (33%) | 19 (37%) | 16 (30%) |
| Pulmonary Disease/COPD, n (%) | 16 (15%) | 19 (19%) | 6 (11%) |
| CKD, n (%) | 18 (17%) | 9 (17%) | 9 (17%) |
| **Laboratory Values and Illness Severity** | | | |
| Creatinine (median, IQR) | 2.0 (1.5, 2.7) | 2.0 (1.5, 2.5) | 2.1 (1.5, 2.8) |
| Lactate (median, IQR) | 3.4 (2.4, 4.7) | 3.8 (2.4, 4.6) | 3.3 (2.5, 5.1) |
| SOFA score (mean ± SD)** | 10.8 ± 3.7 | 10.6 ± 3.7 | 11.0 ± 3.7 |
| Mechanical ventilation, n (%) | 69 (65%) | 35 (67%) | 34 (63%) |

*BMI missing in 2 thiamine patients

**SOFA missing in 4 placebo patients and 3 thiamine patients

6 patients did not have baseline thiamine level

Table S2. Summary of outcomes for the non-thiamine deficient cohort.

| **Thiamine >=8** | | | | | |
| --- | --- | --- | --- | --- | --- |
|  | **Thiamine (n=52)** | **Placebo (n=54)** | | **Geometric mean difference* (95% CI)** | **p-value** |
| **Serum Creatinine (median (IQR), mg/dL)** | | |  | | |
| Enrollment | 2.0 (1.5, 2.5) | 2.1 (1.5, 2.8) | | 0.91 (0.73-1.15) | 0.444 |
| 24 hours | 1.9 (1.2, 2.9) n=51 | 2.2 (1.3, 3.0) n=53 | | 0.87 (0.69-1.10) | 0.242 |
| 48 hours | 2.0 (0.9, 3.0) n=48 | 2.0 (1.2, 3.7) n=53 | | 0.84 (0.67-1.06) | 0.137 |
| 72 hours | 2.0 (0.9, 2.9) n=49 | 2.0 (1.1, 3.9) n=50 | | 0.81 (0.65-1.03) | 0.081 |
| **Serum Lactate (mmol/L, median, IQR)** | | | | | |
| Enrollment | 3.8 (2.4, 4.6) | 3.3 (2.5, 5.1) | | 1.05 (0.79-1.39) | 0.727 |
| 24 hours | 2.2 (1.4, 3.1) n=50 | 2.6 (1.6, 4.4) | | 0.85 (0.64-1.13) | 0.274 |
| 48 hours | 1.9 (1.2, 3.0) n=47 | 2.0 (1.5, 3.0) n=53 | | 0.98 (0.73-1.30) | 0.870 |
| 72 hours | 1.6 (1.1, 2.8) n=46 | 1.8 (1.3, 2.4) n=51 | | 1.02 (0.76-1.35) | 0.912 |
| In-Hospital Survival, n (%) | 31 (60%) | 32 (59%) | | aOR: 0.99 (0.45-2.16) | 0.972 |
| No RRT, n (%) | 46 (88%) | 42 (78%) | | aOR: 2.40 (0.81-7.11) | 0.113 |
| Alive and RRT Free, n (%) | 29 (56%) | 29 (54%) | | aOR: 1.06 (0.49-2.30) | 0.879 |
